# Prolonged myopathy and musculoskeletal symptoms following filgrastim in a 43-Year-Old female stem cell donor: a case-based review

**DOI:** 10.1007/s00296-025-06029-5

**Published:** 2025-11-19

**Authors:** Jagoda Rogowska, Radosław Kakowski, Joanna Makowska, Olga Brzezińska

**Affiliations:** Department of Rheumatology, Medical University of Lodz, Lodz, Poland

**Keywords:** Filgrastim, Myopathy, G-CSF, Drug-induced toxicity, Iatrogenic disease

## Abstract

Filgrastim, a granulocyte colony-stimulating factor (G-CSF), is widely used for the mobilization of peripheral blood stem cells in donors and is generally considered safe. While transient side effects such as bone pain and arthralgia are well-documented, chronic musculoskeletal complications are rarely reported, particularly in otherwise healthy individuals. Herein, we describe the case of a 43-year-old physically active female who developed prolonged musculoskeletal symptoms following stem cell mobilization with filgrastim. The patient initially experienced severe flu-like symptoms and diffuse myalgia, which progressed post-collection and significantly impaired daily function. Despite partial spontaneous improvement, symptoms recurred after physiotherapy and persisted nine months later, prompting hospital admission. Clinical findings included joint pain, swelling, and restricted shoulder mobility. Laboratory tests showed elevated inflammatory markers and positive ANA, while creatine kinase levels remained normal. Imaging revealed Klippel-Feil syndrome and mild degenerative changes. A single dose of intramuscular betamethasone led to gradual recovery of muscular function. This case underscores the importance of recognizing potential long-term iatrogenic effects of G-CSF, even in healthy donors, and highlights the need for individualized follow-up and management.

## Introduction

Filgrastim is a granulocyte-colony stimulating factor widely used for the mobilization of peripheral blood stem cells in healthy donors as part of standard protocols for allogeneic stem cell transplantation [1]. While generally well-tolerated and considered safe, filgrastim administration may lead to a range of adverse effects, predominantly musculoskeletal in nature [3]. These are often transient and self-limiting; however, in some cases, the symptoms may be prolonged and significantly affect quality of life and physical functioning.

This case is unique due to the chronicity and severity of musculoskeletal symptoms in an otherwise healthy, physically active donor, with persistent complaints lasting over nine months after filgrastim administration. Despite normal creatine kinase levels and nonspecific imaging findings, the patient exhibited clinically significant functional impairment. This report adds to the limited body of literature documenting long-term iatrogenic musculoskeletal toxicity of G-CSF in healthy individuals, highlighting the need for increased awareness, post-donation monitoring, and individualized therapeutic approaches in donor follow-up care.

## Case report

A 43-year-old physically active female was admitted to the rheumatology department for evaluation of persistent musculoskeletal pain, joint swelling, and functional limitation. Seven months earlier the patient received granulocyte-colony stimulating factor (filgrastim) as part of peripheral blood stem mobilization for donation. Approximately two weeks prior to mobilization procedure, she experienced an unresolved urinary tract infection. During the course of filgrastim administration, she developed flu-like symptoms, including diffuse myalgia and arthralgia. These symptoms intensified following the stem cell collection and persisted after discontinuation of filgrastim. The patient reported progressive muscle weakness and significant difficulty with ambulation, which gradually improved over the following months without specific treatment. Initially, she did not seek medical attention, being aware of such symptoms as side effects to G-CSF administration. After 2–3 months after donation, the musculoskeletal symptoms slowly subsided. Nonsteroidal anti-inflammatory drugs (NSAIDs) were used without therapeutic benefit. A month later, she completed a course of outpatient physical therapy, after which the symptoms recurred with greater intensity.

At the time of hospital admission, she reported bilateral shoulder pain – predominantly right-sided – elbow pain and swelling of the hands with subjective warming. Physical examination revealed localized swelling of the second metacarpophalangeal joint and dorsum of the left hand, tenderness over both elbow joints (with a noticeable mobile nodule noted on the left), and restricted external rotation in both shoulders. Muscle weakness was absent.

Antinuclear antibodies (ANA) were tested and found to be positive in low titre with a speckled pattern. However, due to limited availability at the time, extended testing for myositis-specific antibodies (e.g., anti-ARS, Mi-2, TIF1γ) was not performed.

Radiographs of the hands, feet, and knees demonstrated mild degenerative changes, and cervical spine imaging revealed Klippel-Feil syndrome at the C5–C6 level, possibly contributing to the upper limb symptoms. Ultrasonography of affected joints did not reveal evidence of active synovitis. ENG was not performed due to limited availability but was recommended on an outpatient basis.

Given the clear temporal association between filgrastim administration and the acute onset of symptoms, drug-induced myopathy was considered the most likely diagnosis. The patient received a single intramuscular dose of betamethasone, which led to partial symptomatic relief. No non-steroidal anti-inflammatory drugs (NSAIDs) were prescribed at discharge.

Upon follow-up several weeks later, the patient reported significant clinical improvement, with no remaining complaints apart from mild discomfort attributable to compression from a cervical rib, which was subsequently surgically removed. Notably, within six months of the initial presentation, all musculoskeletal symptoms resolved completely. She was discharged in good general condition, with recommendations for further follow-up and supportive care.

MRI of the affected muscles was not conducted, as the patient showed a rapid and complete clinical response to short-term corticosteroid therapy, which led to discontinuation of further diagnostics.

**Fig. 1:**
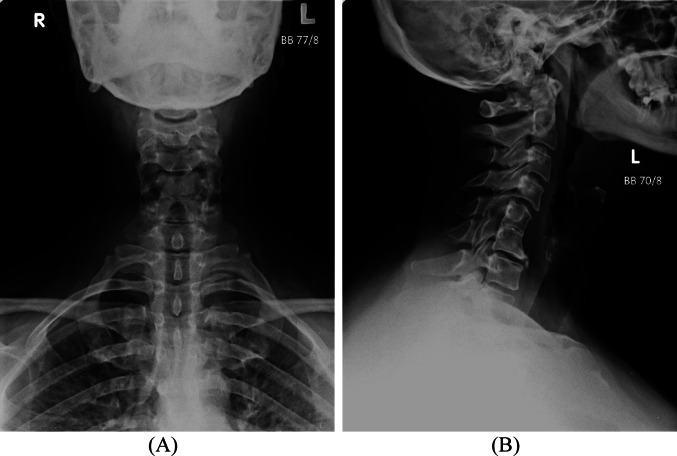
**A-B **Anteroposterior (**A)** and lateral (**B**) radiographic views of the cervical spine demonstrating Klippel-Feil syndrome at the C5–C6 level, characterized by congenital fusion of the vertebral bodies with loss of normal intervertebral disc space and reduced cervical mobility

Needless to say, ultrasound (US) may reveal musculoskeletal soft tissue pathologies more accurately and often correlates better with the clinical presentation than magnetic resonance imaging (MRI). In our patient, US findings (significant fluid/tendinosis) were more consistent with the severe clinical picture than MRI (mild tendinosis) and allowed us to distinguish tendinitis from possible triangular fibrocartilage complex pathology. In such cases, when modalities like electromyography or MRI are not available, US emerges as a reliable, accessible, and convenient diagnostic tool, underscoring its pivotal role in musculoskeletal evaluation [17] (Fig. 1).

Given the full and sustained resolution of symptoms following glucocorticoid treatment and no recurrence during outpatient follow-up (the patient remains under the clinic’s care), the presence of an immune-mediated inflammatory myopathy is highly unlikely. The clinical presentation was considered most consistent with a filgrastim-associated, self-limiting myopathy rather than classic idiopathic inflammatory myositis or polymyalgia rheumatica.

Laboratory tests were presented in Table 1.

**Table 1:** Summarized immunological and inflammatory markers in the patient

Test	Result	Reference range	Interpretation
ESR (erythrocyte sedimentation rate)	21 mm/h	< 12 mm/h	Slightly elevated
CRP (C-reactive protein)	6,6 mg/l	< 6,0 mg/l	Elevated
CK (creatine kinase)	138 U/l	45,0–84,0 µmol/l	Normal
ANA (antinuclear antibodies)	1:320	< 1:160	Significantly positive
RF (rheumatoid factor)	< 10,0 IU/ml	< 14,0 IU/ml	Negative
Anti-CCP antibodies	0,769 RU/ml	< 5,00 RU/ml	Negative

## Search strategy

A systematic literature search was conducted using three scholarly databases: **PubMed/MEDLINE**, **Web of Science**, and **Scopus**, covering the period from **2010 to 2023**. The search aimed to identify articles relevant to musculoskeletal and neuromuscular adverse effects associated with **filgrastim**, **pegfilgrastim**, and related granulocyte colony-stimulating factors (G-CSFs). Additional sources were identified by manually screening the reference lists of included articles.

Keywords used in the search included: “filgrastim,” “pegfilgrastim,” “granulocyte colony-stimulating factor,” “drug-induced myopathy,” “rhabdomyolysis,” “statin-associated muscle symptoms,” and “fluoroquinolone-induced tendinopathy.” Boolean operators were applied to combine search terms as follows: (“filgrastim” AND “rhabdomyolysis”) OR (“pegfilgrastim” AND “myopathy”) OR (“statins” AND “muscle symptoms”) OR (“fluoroquinolones” AND “tendinopathy”) OR (“drug-induced” AND “myopathy”).

The initial search yielded **246 articles**. After removal of duplicates and screening of titles and abstracts, **230 records** were assessed. Of these, **62 full-text articles** were reviewed for eligibility. Based on predefined criteria, **46 articles** were excluded due to irrelevant content, lack of clinical detail, or focus solely on oncologic outcomes. Ultimately, **16 articles**met the inclusion criteria and were included in the final review. Two additional studies were identified through reference list screening, bringing the total to **18 included articles**.

## Discussion

Peripheral blood stem cell mobilization using granulocyte-colony stimulating factors (G-CSF) remains a cornerstone of both allogeneic and autologous transplantation protocols [1]. Filgrastim, a recombinant G-CSF, is widely used due to its efficacy and well-documented safety profile. While the most common adverse events associated with filgrastim include bone pain, fatigue, and low-grade fever [2], more serious complications have been reported, including splenic rupture, acute lung injury, and severe musculoskeletal symptoms [3].

Prolonged myopathy following G-CSF administration, particularly in healthy donors, is a rare and underrecognized adverse effect. In our case, the patient developed persistent musculoskeletal pain, joint swelling, and functional limitation beginning shortly after filgrastim administration. The clear temporal association between the initiation of filgrastim and the onset of symptoms, coupled with the absence of other evident causes, supports the hypothesis of filgrastim-induced musculoskeletal toxicity.

This case differs from previously reported acute complications such as rhabdomyolysis or splenic rupture [4], as it represents a prolonged and functionally disabling course of symptoms, gradually resolving over several months. The recurrence of symptoms following physiotherapy and only partial response to NSAIDs further supports the presence of a chronic, low-grade inflammatory or immune-mediated process. A single intramuscular dose of betamethasone resulted in symptomatic relief, indicating a potential inflammatory component.

Muscle pain and stiffness are known adverse effects of several commonly used medications, often presenting as part of a spectrum ranging from benign myalgias to true drug-induced inflammatory myopathies. Among the most frequently implicated drug classes are statins, with myalgia reported in up to 10–20% of patients, though true statin-induced myopathy is much rarer, affecting approximately 0.1–0.5% [7].

Recent studies have highlighted the role of genetic factors in the pathogenesis of statin-associated muscle symptoms (SAMS), ranging from benign myalgias to severe rhabdomyolysis. Among the most studied genetic variants is the SLCO1B1 c.521T >C polymorphism (rs4149056), which affects the hepatic uptake of statins by altering the function of the OATP1B1 transporter. This results in increased systemic exposure to statins and a correspondingly higher risk of muscle toxicity, particularly in patients treated with simvastatin. Genome-wide association studies have consistently confirmed this association [8]. What is more, polymorphisms in genes involved in drug metabolism, such as CYP3A4 and CYP2C9, may influence plasma concentrations of statins, thereby modifying the risk of adverse muscular effects [9]. Furthermore, variants in mitochondrial genes, including POLG and COQ2, have been implicated in impaired energy metabolism and increased vulnerability to statin-induced mitochondrial dysfunction [10]. These findings underscore the importance of considering genetic factors when evaluating patients for statin therapy, as it may predispose individuals to adverse muscular effects.

Other agents associated with musculoskeletal side effects include antiretroviral drugs (particularly zidovudine), interferons, immune checkpoint inhibitors, and certain antibiotics such as fluoroquinolones, which may cause tendonitis and myopathy, particularly in elderly or corticosteroid-treated patient [11, 12]. The exemplary substances and drugs that should be exanimated for in case of occurrence of painful myopathy has been indicated in Table 2.

G-CSFs are less commonly linked with chronic or delayed musculoskeletal toxicity. While bone pain occurs in up to 30–70% of filgrastim-treated patients, especially during stem cell mobilization [13], these symptoms are typically self-limited and resolve shortly after discontinuation. Prolonged or delayed-onset musculoskeletal symptoms, such as those observed in our case, are rarely reported and likely underrecognized, particularly in healthy donors who are otherwise asymptomatic prior to G-CSF exposure.

Importantly, G-CSFs have also been implicated in immune-mediated adverse events, including large-vessel vasculitis (LVV). Several case reports and reviews have identified G-CSF—particularly pegfilgrastim—as a potential trigger for LVV, manifesting as aortitis or arteritis, often accompanied by systemic symptoms such as fever, malaise, and elevated inflammatory markers. This is especially relevant in the context of polymyalgia rheumatica (PMR), which may overlap clinically with G-CSF-induced vasculitis and present with proximal muscle pain and stiffness [18]. The immune-stimulatory properties of G-CSF are believed to promote a proinflammatory cytokine milieu and neutrophil activation, potentially contributing to vascular inflammation and musculoskeletal injury [19].

**Table 2:** Drugs associated with muscle pain or myopathy [14]

Drug/drug class	Typical muscular side effects	Frequency
Statins	Myalgia, myopathy, rarely rhabdomyolysis	Myalgia: 10–20%; Myopathy: ~0.1–0.5%
G-CSF (e.g., Filgrastim)	Bone pain, myopathy, prolonged pain in rare cases	Bone pain: 30–70%; myopathy: rare
Zidovudine (antiretroviral)	Myopathy, muscle weakness	Uncommon
Interferons	Myalgia, inflammatory myopathy	Uncommon to rare
Fluoroquinolones	Tendinitis, tendon rupture, myopathy	Rare, but ↑ risk with steroids
Immune checkpoint inhibitors	Autoimmune myositis, inflammatory syndromes	Rare
Levodopa [15]	Generalized dyskinesia, drowsiness, mild rigidity	Common complication in patients with Parkinson’s disease
Cocaine [16]	Myalgia, muscle weakness, massive rhabdomyolysis, acute renal failure	More common after intravenous use or after smoking the alkaloid freebase

In our case, the patient presented with persistent musculoskeletal pain, stiffness, and inflammatory features lasting several months after filgrastim exposure, without evidence of overt necrotizing myopathy or vasculitis on imaging. While acute inflammatory muscle injury, including necrotizing myositis, has been reported in association with G-CSF [5], our findings are more consistent with a non-necrotic, protracted inflammatory musculoskeletal syndrome. Notably, the patient’s positive ANA, elevated ESR and CRP, and recurrence of symptoms following physiotherapy raise the possibility of immune activation or subclinical vasculitic involvement, although definitive imaging for large-vessel vasculitis was not performed.

What is more, other possible diagnosis that may have been recognized is complex regional pain syndrome (CRPS), which particularly presents similar symptoms, concerning decreased range of motion, weakness in the affected limbs and severe pain. However, contrary to myopathy after filgrastim, complex regional pain syndrome is a localized, asymmetrical post-traumatic neuropathic pain condition with sensory, autonomic and trophic changes but without CK elevation [20]. Therefore, CRPS was not considered, as the patient’s symptoms were generalized, symmetrical, and associated with elevated CK, which are typical for filgrastim-induced myopathy.

This case underscores the importance of recognizing the broader immunologic and rheumatologic spectrum of G-CSF-induced toxicity—including vasculitis, autoimmune manifestations, and delayed-onset myopathy—even in previously healthy donors. Clinicians should maintain a high index of suspicion for G-CSF-related complications in the post-donation period, especially when symptoms are atypical, prolonged, or recur after physical rehabilitation. Early recognition and appropriate evaluation may prevent long-term functional impairment and guide more personalized donor follow-up strategies [6].

## Conclusions

Filgrastim is a cornerstone in the mobilization of peripheral blood stem cells and is generally well tolerated in healthy donors. However, this case highlights that even in otherwise healthy individuals, filgrastim can lead to prolonged, functionally limiting musculoskeletal adverse effects resembling drug-induced myopathy. The strong temporal association between drug administration and symptom onset, the absence of alternative explanations, and response to corticosteroids support the diagnosis of filgrastim-induced musculoskeletal toxicity. Awareness of such atypical presentations is essential for early recognition, appropriate management, and prevention of unnecessary diagnostic delays. Clinicians should maintain a high index of suspicion in donors presenting with persistent pain or weakness following G-CSF administration and provide adequate follow-up care, even in the absence of laboratory signs of muscle damage.

## Data Availability

This case report is an original work and has not been copied or published elsewhere, in whole or in part, in any language.

## Declarations

### Editing agencies involved and the use of AI or editing software

This paper has been prepared by the authors without the involvement of editing agencies. No AI technologies or editing software were utilized in the creation of this work.
